# Metabolic syndrome related to cardiovascular events in a 10-year prospective study

**DOI:** 10.1186/s13098-015-0096-2

**Published:** 2015-11-19

**Authors:** Laura Kazlauskienė, Jūratė Butnorienė, Antanas Norkus

**Affiliations:** Institute of Endocrinology, Lithuanian University of Health Sciences, Eiveniu 2, 50009 Kaunas, Lithuania

**Keywords:** Metabolic syndrome, Cardiovascular events, Metabolic syndrome components

## Abstract

**Background:**

Metabolic syndrome (MetS) becomes a serious society health problem. The main risk factors of MetS are related to the increased risk of cardiovascular diseases, appearance of stroke, type 2 diabetes mellitus and the growing risk of mortality. MetS stimulates the appearance of early atherosclerosis, its progress and accelerates the frequency of cardiovascular complications related to atherosclerosis and diabetes mellitus.

**Objective:**

To evaluate the risk of cardiovascular events (myocardial infarction, stroke) among the individuals with MetS in a 10 year prospective study; to identify MetS components that determine risk and character of cardiovascular events.

**Methods:**

The study design was prospective. It was started in 2003 to assess the risk factors, clinical components, diagnostic criteria of MetS. At the second stage in 2013 the individuals were repeatedly invited to evaluate cardiovascular pathology that was confirmed by cardiologist and neurologist. The 45 years old and older citizens of Lithuanian district participated in the study. 1115 individuals (562 men and 553 women) were randomly selected in 2003. 538 respondents: 278 (51.70 %) men and 260 (48.30 %) women participated in the repeated study in 2013.

**Results:**

During the study myocardial infarction (MI) was confirmed to 7.43 % individuals taken part in the study, stroke—to 4.28 % individuals. The odds’ ratio (OR) of MI between individuals with MetS and without MetS was 1.80 (95 % CI 1.67–1.97), p < 0.05. The OR of stroke for individuals with MetS and without MetS was 2.05 (95 % CI 1.21–2.54), p < 0.05. The OR of MI between men with abdominal obesity and identified MetS was 3.12 (95 % CI 2.77–3.53), p < 0.05. The OR of stroke between men with low level of high density lipoprotein cholesterol and identified MetS was 4.98 (95 % CI 4.40–5.65), p < 0.05. The OR of stroke between men with hypertriglyceridemia and identified MetS was 8.43 (95 % CI 7.45–9.54), p < 0.05.

**Conclusions:**

Individuals with identified MetS have 1.80 and 2.05 times higher statistically significant probability, respectively, for MI and stroke events, than individuals without MetS. Separate components or MetS increase risk of cardiovascular events in men: abdominal obesity increases risk of MI, and low level of high density lipoprotein cholesterol and hypertriglyceridemia increase risk of stroke.

## Background

Cardiovascular diseases remain the leading cause of the death in industrialised countries and in many of the developing countries in parallel with industrialisation, urbanisation and changes of life style. The increase of cardiovascular events in economically developed countries determine the risk factors of coronary heart disease (CHD): hypertension, abdominal obesity, dyslipidemia, failure of glucose metabolism that are caused by sedentary lifestyle, dietary changes, stress, smoking and elder age. MetS, connecting 3–5 risk factors of CHD, increases frequency of cardiovascular events especially among the elderly population [1]. Some studies state that MetS is more frequent 1.50–2.00 times to individuals with CHD. MetS significantly increases frequency of cardiovascular events, their progress and risk of consequences [2, 3]. The risk of CHD is 7 times higher among individuals with MetS and diagnosed diabetes mellitus [4].

Studying the relation between MetS and increased risk of CHD among the 40-year old individuals (more than 1/3 of individuals with hypertension or low high density lipoprotein cholesterol (HDLP)) there was identified that risk to develop CHD is higher to individuals with MetS (p < 0.001) [5]. Among the individuals participated in the study, 55 had 10 % higher risk to get cardiovascular event in a 10-year time, and MetS was identified to 33 (60 %) of them. According to the study data, individuals with identified hypertriglyceridemia had a higher risk of cardiovascular events or there were more risk factors to the individuals with increased low density lipoprotein cholesterol (LDLP) level (p < 0.05).

According to the data of epidemiological and clinical studies, separate MetS component is independent risk factor of CHD [6–9]. Some studies analyse the relation between separate MetS components and risk of cardiovascular diseases [10–12]. Influence of separate MetS components to development of CHD differs but the greater number of MetS components leads to the higher risk of CHD [12]. On the base of Lithuanian study data, the OR of CHD to individuals with 4–5 components and without any component of MetS is 7.20 (95 % CI 2.05–26.6) in men’s group and 4.14 (95 % CI 1.70–10.2) in women’s group; comparing the individuals with 4–5 components and 1–3 components, the OR was 2.32 (95 % CI 1.01–5.23) in men’s group and 1.76 (95 % CI 0.98–3.15) in women’s group [13].

### The study aim

To evaluate the risk of cardiovascular events to individuals with identified MetS in 10-year time; to clarify the MetS components leading risk and character of cardiovascular events.

## Methods

The study has been approved by Kaunas Regional Bioethics Committee, Lithuania (No. BE-2-34/2011, No. PI-22/2013).

The study was started in 2003 to evaluate the risk factors, clinical components, diagnostic criteria of MetS. 1115 (562 men and 553 women) adults of permanent population (living in town and village)from Lithuanian district were randomly selected to participate in the study at the first stage in 2003.

In 2011 year 966 invitations were sent to participate at the second stage of the study. The invitations were sent by post-office. 10.40 % individuals from the starting point of the study had died, 2.96 % had moved to other living places. 538 (278 men and 260 women, accordingly 51.70 % and 48.30 %) individuals participated at the second stage in 2011–2013. The rate of responses was 55.70 %. Individuals were asked to arrive for testing without taking any food for at least 12 h. Individuals presented the written agreement to participate in the study. The questionnaire with demographic, life and health history data was filled, assessed the objective clinical condition, anthropometric data, blood pressure measured, fasting glycaemia and lipids panel were assessed by laboratory tests.

Height and weight of all the participants were measured. Both body mass and height were measured using standardised and calibrated medical electronic scales and height meter. Body mass index (BMI) was estimated using the following formula: weight, kg/height, m^2^. Waist circumference was measured. Measurements were performed at median line of waist between the lower rib margin and wing of ileum was measured while the patient held his/her breath. Arterial blood pressure (ABP) was measured according to recommendations for correct ABP measurement based on the Guidelines of the European Society of Hypertension, 2007 [14]. After the patient was seated for approximately 5 min in quiet environment, standard cuff was put on his forearm at heart level. To assess the circulation of carbohydrates there was done a fasting glucose test of vein blood plasma using the means of biochemical analyser BIOSEN C–line. HDLP cholesterol was measured using CHOD-PAP ferment photometric method; triglycerides—GPO–PAP were measured using ferment photometric method [15, 16].

MetS was assessed according to National Cholesterol Education Program III modified criteria [17]. Subjects were included in MetS group when at least three symptoms of listed below were confirmed:

1. Waist circumference in men >102 cm, in women >88 cm;
2. Systolic blood pressure ≥130 mm Hg and/or diastolic blood pressure ≥85 mm Hg or anti hypertensive medications are used;
3. Blood glucose level (fasting) ≥5.6 mmol/l;
4. Triglycerides (TG) ≥1.7 mmol/l or special treatment to lower TG level is applied;
5. HDLP cholesterol <1.03 mmol/l in men and <1.29 mmol/l in women.

MI was identified by cardiologist on the base of clinical angina syndrome, newly appeared typical ischaemic changes in electrocardiogram and increase of troponin I concentration.

Ischaemic stroke was confirmed by neurologist on the base of clinical features and head brain computer tomography scan.

### Statistical analysis

Statistical analysis was performed using the Statistical Package for Social Sciences (SPSS v. 20.0). The data was presented as average, standard deviation. To compare the differences between groups with normal distribution Student t test was used. To compare the differences between averages when distribution was not normal or the group was less than 20 Mann–Whitney U test was used. To compare frequency in different groups Chi-square (precision Fisher criterion) criterion was used. The probability of a certain variable, in case of a certain factor, was evaluated by calculating the odds ratio (OR) and its 95 % confidence interval (CI) using the one way logistic regression analysis. The significant level of p < 0.05 was checked as statistical significant.

## Results

### Clinical characteristics of the individuals taken part in the study

538 individuals have taken part in the repeated study: 278 (51.70 %) men and 260 (48.30 %) women, p > 0.05. The main characteristics of the individuals are presented in the Table 1. The age of individuals was 55–92 and there was not significant difference of the age in men and women’s groups. Due to genders’ constitutional peculiarities, anthropometric data of men and women was significantly different. Comparing genders, obesity among the women was more frequent. MetS was identified on the base of National Cholesterol Education Program Adult Treatment Panel III (NCEP/ATP III). According to the data of analysis and objective investigation of individuals, MetS was identified to 43.90 % individuals: 18.60 % men and 25.30 % women, p < 0.05. According to the data of the study MetS was more frequent among the women.

**Table 1:** Characteristics of the study sample

Characteristics	Men, n = 278	Women, n = 260	p
Age (years), m ± SD	70.73 ± 8.56	70.35 ± 8.3	>0.05
Height (cm)	170.31 ± 6.78	158.31 ± 17.22	<0.05
Weight (kg)	82.80 ± 14.82	77.05 ± 16.13	<0.05
BMI (kg/m^2^)	28.50 ± 4.49	31.05 ± 5.91	<0.05
Waist circumference (cm)	101.58 ± 13.24	97.28 ± 14.22	<0.05
SBP (mmHg)	151.54 ± 21.24	152.73 ± 19.57	>0.05
DBP (mmHg)	88.12 ± 9.51	88.76 ± 10.48	>0.05
Fasting blood glucose (mmol/l)	4.9 ± 0.70	4.81 ± 0.71	<0.05
HDLP cholesterol (mmol/l)	1.4 ± 0.50	1.42 ± 0.44	<0.05
TG (mmol/l)	1.28 ± 0.92	1.3 ± 0.62	<0.05
Frequency of MetS, n (%)	100 (35.97)	136 (52.31)	<0.05
Cardiovascular events (MI and stroke), n (%)	35 (12.59)	28 (10.77)	>0.05
Frequency of MI, n (%)	21 (7.55)	19 (7.31)	>0.05
Frequency of stroke, n (%)	14 (5.04)	9 (3.46)	<0.05*

*n* the number of tested individuals, *m* average, *SD* standard deviation, *p* significance level, *BMI* body mass index, *SBP* systolic blood pressure, *DBP* diastolic blood pressure, *MetS* metabolic syndrome, *TG* triglycerides, *MI* myocardial infarction, *HDLP* high density lipoprotein cholesterol

* p the exact Fischer test

### Frequency of cardiovascular events among the individuals taken part in the study

In a 10 year period MI was confirmed to 7.43 % individuals, stroke diagnosis was confirmed to 4.28 %. According to the study data MI is more frequent among the individuals with MetS than without MetS, respectively 9.75 vs. 5.63 %, p > 0.05. Stroke was confirmed more often to individuals with MetS than to ones without MetS, accordingly 5.93 vs. 2.98 %, p > 0.05. Comparing the frequency of MI among the men and women with MetS, the pathology was confirmed more frequent to the women than men, accordingly 10.30 vs. 9.00 %, p > 0.05. Comparing the frequency of stroke in the similar group of individuals (men and women with identified MetS), the significant difference was not observed, 5.15 vs. 7.00 %, p > 0.05. Analysing the data of MI, the frequency among the different genders’ individuals without MetS, MI was confirmed more often to men than women, respectively 7.70 vs. 5.44 %, p > 0.05. Stroke was confirmed more often to men than women, respectively 4.49 vs. 2.18 %, p > 0.05. However, difference was of no statistical significance, probably due to too few cases.

The OR of MI between individuals with MetS and without MetS was 1.80 (95 % CI 1.67–1.97), p < 0.05. The OR of stroke for individuals with MetS and without MetS was 2.05 (95 % CI 1.21–2.54), p < 0.05.

Using the method of logistic regression there was evaluated the relation between MetS components and cardiovascular events (Tables 2, 3).

**Table 2:** OR of MI to individuals with identified MetS or an only component of MetS

Components of MetS	Men with confirmed MetS, OR, [95 % CI], p	Women with confirmed, MetS, OR, [95 % CI], p
Waist circumference	3.12 [2.77–3.53], 0.049	4.36 [3.84–4.96], 0.131
Hypertension	0.52 [0.47–0.59], 0.253	0.92 [0.81–1.05], 0.916
Hyperglycaemia	2.62 [2.32–2.96], 0.367	–
Low level of high density lipoprotein cholesterol	1.36 [1.21–1.54], 0.649	3.09 [2.72–3.51], 0.071
Elevated triglycerides	1.69 [1.50–1.91], 0.390	1.05 [0.93–1.20], 0.935

*OR* odds ratio, *p* significance level, *CI* confidence interval, *MetS* metabolic syndrome

**Table 3:** OR of stroke when MetS or an only its component is identified

Components of MetS	Men with confirmed MetS, OR, [95 % CI], p	Women with confirmed MetS, OR, [95 % CI], p
Waist circumference	2.50 [2.21–2.83], 0.281	1.74 [1.54–1.99], 0.618
Hypertension	–	0.82 [0.72–0.94], 0.858
Hyperglycaemia	6.80 [6.01–7.70], 0.057	4.06 [3.57–4.62], 0.190
Low level of high density lipoprotein cholesterol	4.98 [4.40–5.64], 0.034	1.35 [1.19–1.54], 0.790
Elevated triglycerides	8.43 [7.45–9.54], 0.004	1.89 [1.67–2.16], 0.482

*OR* odds ratio, *p* significance level, *CI* confidence interval, *MetS* metabolic syndrome

To men with waist circumference >102 cm probability of MI is 3.12 times higher (95 % CI 2.77–3.53) than with identified MetS, p < 0.05.

Probability of MI to men with identified hyperglycaemia and identified MetS is 2.62 (95 % CI 2.32–2.96), p > 0.05. However, difference was not statistically significant.

Probability of MI to women with waist circumference > 88 cm and identified MetS is 4.36 (95 % CI 3.84–4.96), p > 0.05.

Probability of MI to women with identified low HDLP cholesterol level (<1.29 mmol/l) and identified MetS is 3.09, p > 0.05.

Probability of stroke to men with identified hypertriglyceridemia is 8.43 times (95 % CI 7.45–9.54) higher than to men with identified MetS, p < 0.05. Probability of stroke to men with identified hyperglycaemia and identified MetS was 6.80 (95 % CI 6.01–7.70), p > 0.05. OR of stroke to men with identified low HDLP cholesterol level (< 1.03 mmol/l) was 4.98 times (95 % CI 4.40–5.64) higher than to men with identified MetS, p < 0.05.

OR of stroke to women with identified hyperglycaemia and identified MetS was 4.06 (95 % CI 3.57–4.62), p > 0.05.

Probability of MI to individuals with increased waist circumference is 1.50 times (95 % CI 1.09–2.08) higher than probability of stroke.

Employing the method of logistic analysis, the correlation between MetS components and cardiovascular pathology in different (men/women) genders was evaluated.

It was evaluated the OR of MI in regard to the number of MetS components between genders. OR of MI to men with 2 MetS components is 1.38 times (95 % CI 1.22–1.57, p = 0.309) higher than to women. OR of stroke to men with 2 MetS components is 1.85 times (95 % CI 1.63–2.10, p = 0.021) higher than to women. OR of stroke to men with 3 MetS components is 1.37 times (95 % CI 1.21–1.56, p = 0.038) higher than to women.

It was evaluated the number of MetS components which influence appearance of cardiovascular events. Analysis was done to both genders separately. The results are presented in the Table 4. Stroke was mostly experienced in men with identified 3 components of MetS—2 (5.89 %) and MI was mostly experienced in men with 4 components, respectively 2 (18.19 %). Women experience cardiovascular events (stroke or MI) in cases with identified 4 components of MetS, respectively 2 and 4 (15.39 vs. 30.77 %).

**Table 4:** Distribution of MetS components which influence apperance of cardiovascular events (stroke, MI) to both genders

Cardiovascular events	MetS with 3 components, n (%) (n = men/women) (n = 34/57)	MetS with 4 components, n (%) (n = men/women) (n = 11/13)	MetS with 5 components, n (%) (n = men/women) (n = 2/2)
Men			
Stroke	2 (5.89)	–	1 (50.00)
MI	2 (5.89)	2 (18.19)	–
Women			
Stroke	2 (3.51)	2 (15.39)	–
MI	4 (7.02)	4 (30.77)	1 (50.00)

*n* the number of tested individuals, *MetS* metabolic syndrome, *MI* myocardial infarction

## Discussion

Recently the new statement, that ageing and MetS have different influence on men and women genders, is appearing more and more often. The different ageing of both men and women blood-vessels’ was acknowledged in 2010 after the Arterial ageing study [18].

According to the data of some studies cardiovascular diseases’ (CVD) risk related to MetS is higher to women than men. It can be accounted for women arteries’ tightness but there is a lack of evidence to confirm the statement. Moreover, the data of different studies are contradictory. The developing tightness of arteries due to MetS could be buttressed up by chronic inflammation and dysfunction of endothel [19–21]. This is probably due to the increased risk of CVD that is typical to the state. Studies ascertained have shown that women are more influenced by MetS to get atherosclerosis [22, 23].

R. Kawamoto and co-authors after researching 388 men and 480 women ascertained that early atherosclerosis among the patients with MetS is identified only to women. However, epidemiology research has shown that men have atherosclerotic plates more often than women [24]. The scientists tend to explain this difference as influence of hormones. During the menopause women lose the influence of oestrogen which reduces atherosclerosis. The deficit in oestrogens for elderly women due to menopause is related to complications of CVD. It is stated that normal physiological concentration of oestrogens prevent elderly men’s heart and blood-vessels’ system. Testosterone in men’s organism produces oestrogens [25]. Therefore, MetS causes negative gender hormones alteration that changes CVD pathogenesis and stimulates early atherosclerosis for both genders [26].

According to the prospective study data, comparing the prevalence of MI among the adults population, the pathology of MI was more frequently confirmed to individuals with MetS. It was confirmed more frequently to women than men, respectively 10.30 vs. 9.00 %, p > 0.05. Comparing the prevalence of stroke in a similar group of individuals, significant difference was not observed, accordingly 5.15 vs. 7.00 %, p > 0.05.

Nowadays there is done meta analysis of 87 studies that involved 951,083 individuals. The results showed that individuals with identified MetS have twice higher risk to develop cardiovascular diseases. Mortality to individuals with identified MetS is higher 1.50 times [27].

According to our study, individuals with identified MetS have 1.80 (95 % CI 1.67–1.97) and 2.05 (95 % CI 1.21–2.54) times higher statistically significant probability, respectively, for MI and stroke events, than individuals without MetS.

So far there is no a generally accepted view on the various risk factors that compound MetS. The risk factors’ aggression, the frequency in combinations are under investigation, it is unclear which component of the combination is more important, which appeared first and caused other factors leading to the occurrence. Many researchers indicate that glucose metabolism disorder raises a higher risk of CHD in combination of MetS components. Insulin resistance and abdominal fat accumulation associated with lipoprotein metabolism disorders (atherogenic dyslipidemia) are consequences of glucose metabolism disorder. According to the data of the prospective study it was evaluated the risk of cardiovascular events by identified/not identified MetS or identified a component of MetS. The OR of MI to men and women with identified abdominal obesity is respectively 3.12 (95 % CI 2.77–3.53) vs. 4.36 (95 % CI 3.84–4.96). It indicates that MetS increases the possibility of MI. The OR of MI to men with identified hyperglycaemia is 2.62 (95 % CI 2.32–2.96). The OR of stroke to women with identified hyperglycaemia is 4.06 (95 % CI 3.57–4.62), to men—6.80 (95 % CI 6.01–7.70). The study results demonstrate that the OR of cardiovascular events (MI or stroke) to individuals with an identified MetS component, especially with identified abdominal obesity or hyperglycaemia is higher than to individuals with confirmed MetS. Hyperglycaemia and abdominal obesity are stronger prognostic indexes in common population than syndrome as unit.

There are some data showing that increase of cardiovascular diseases is related directly with a greater number of syndrome components [10–12].

Beaver Dam study tested 4423 individuals aged 43–86 without MetS at the starting point. The study ascertained the influence of MetS components to the risk of cardiovascular disease: the more components, the higher risk. The risk of cardiovascular diseases increased 6 times during 5 years period to individuals with identified 4 or more components [11]. In Japan, after researching 6182 men aged 35–59 without confirmed cardiovascular disease at a starting point of the study, the risk of CVD during 7 years of the research significantly increased as it was caused by increase of components number. The OR of CVD to men with an identified MetS component was 4.98; 5.86; 9.98; p < 0.001 [12].

According to the prospective study data, stroke was mostly experienced in men with identified 3 components of MetS and MI was mostly experienced in men with 4 components. Women experience cardiovascular events (stroke or MI) in cases with identified 4 components of MetS.

Large long-term (12-year observed 62,000 men and 262,000 women) studies’, conducted in the United States, data were used to estimate the relationship between common and cardiovascular mortality and the BMI [28].

An obvious increase in total mortality was observed at a BMI over 25 kg/m^2^. CVD drastic increase in the mortality was observed with BMI over 30 kg/m^2^. Gothenburg studies made in Sweden focused on the position of fat in the body, i.e. waist/hip ratio in touch with MI and stroke frequency. The results confirmed the hypothesis—MI and frequency of stroke among the patients with abdominal obesity was higher [29].

According to the study data, the OR of MI to individuals with increased waist circumference is 1.50 times (95 % CI 1.09–2.08) higher.

The significant influence of diabetes mellitus in MetS combinations indicates the fact that 65 % patients die from CVD, while MetS is identified to 66–86 % of individuals with diagnosed diabetes mellitus [30–33].

According to Kaunas Medical University Clinics’ Institute of Cardiology conducted research, MetS was identified to more than half patients with acute coronary syndrome. The most common components of MetS were the hypertension and abdominal obesity. There were identified one or two components of MetS to more than 1/3 (36.50 %) patients with acute coronary syndrome; Combination of 3 components of MetS was identified to 1/3 (29.20 %) of patients. Combination of 4–5 components was identified to 1/3 (30.40 %) of patients. No one component of MetS was not identified to only 3.90 % of patients. Hypertension and abdominal obesity were identified in more than a half of events (57.80 %) and it was found in 2–5 components combinations [34].

According to the study, hyperglycaemia and hypertriglyceridemia were the most frequent components of MetS among the individuals who experienced cardiovascular events. There was no significant difference between genders.

The study approved that MetS increases CVD risk. It is very important to identify MetS in time and evaluate the dominant component, and employ adequate non-pharmaceutical and pharmaceutical treatment. Appropriate, adequate, individual treatment of MetS will prevent serious chronic diseases such as diabetes type 2 and CVD occurrence.

## Conclusions

1. Individuals with identified MetS have 1.80 (95 % CI 1.67–1.97) and 2.05 (95 % CI 1.21–2.54) times higher statistically significant probability, respectively, for MI and stroke events, than individuals without MetS.
2. Separate components or MetS increase risk of cardiovascular events in men: abdominal obesity increase risk of MI, and low level of high density lipoprotein cholesterol and hypertriglyceridemia increase risk of stroke.
